# Reactive oxygen species generation mediated by NADPH oxidase and PI3K/Akt pathways contribute to invasion of *Streptococcus agalactiae* in human endothelial cells

**DOI:** 10.1590/0074-02760170421

**Published:** 2018-04-05

**Authors:** Jessica Silva Santos de Oliveira, Gabriela da Silva Santos, João Alfredo Moraes, Alessandra Mattos Saliba, Thereza Christina Barja-Fidalgo, Ana Luíza Mattos-Guaraldi, Prescilla Emy Nagao

**Affiliations:** 1 Universidade do Estado do Rio de Janeiro, Instituto de Biologia Roberto Alcântara Gomes, Departamento de Biologia Celular, Laboratório de Biologia Molecular e Fisiologia de Estreptococos, Rio de Janeiro, RJ, Brasil; 2 Universidade do Estado do Rio de Janeiro, Instituto de Biologia Roberto Alcântara Gomes, Departamento de Biologia Celular, Laboratório de Farmacologia Celular e Molecular, Rio de Janeiro, RJ, Brasil; 3 Universidade do Estado do Rio de Janeiro, Faculdade de Ciências Médicas, Rio de Janeiro, RJ, Brasil

**Keywords:** Streptococcus agalactiae, HUVEC, reactive oxygen species, NADPH oxidase, p47*phox*, PI3K/Akt pathway

## Abstract

**BACKGROUND:**

*Streptococcus agalactiae* can causes sepsis, pneumonia, and meningitis in neonates, the elderly, and immunocompromised patients. Although the virulence properties of *S. agalactiae* have been partially elucidated, the molecular mechanisms related to reactive oxygen species (ROS) generation in infected human endothelial cells need further investigation.

**OBJECTIVES:**

This study aimed to evaluate the influence of oxidative stress in human umbilical vein endothelial cells (HUVECs) during *S. agalactiae* infection.

**METHODS:**

ROS production during *S. agalactiae*-HUVEC infection was detected using the probe CM-H2DCFDA. Microfilaments labelled with phalloidin-FITC and p47*phox*-Alexa 546 conjugated were analysed by immunofluorescence. mRNA levels of p47*phox* (NADPH oxidase subunit) were assessed using Real Time qRT-PCR. The adherence and intracellular viability of *S. agalactiae* in HUVECs with or without pre-treatment of DPI, apocynin (NADPH oxidase inhibitors), and LY294002 (PI3K inhibitor) were evaluated by penicillin/gentamicin exclusion. Phosphorylation of p47*phox* and Akt activation by *S. agalactiae* were evaluated by immunoblotting analysis.

**FINDINGS:**

Data showed increased ROS production 15 min after HUVEC infection. Real-Time qRT-PCR and western blotting performed in HUVEC infected with *S. agalactiae* detected alterations in mRNA levels and activation of p47*phox*. Pre-treatment of endothelial cells with NADPH oxidase (DPI and apocynin) and PI3K/Akt pathway (LY294002) inhibitors reduced ROS production, bacterial intracellular viability, and generation of actin stress fibres in HUVECs infected with *S. agalactiae*.

**CONCLUSIONS:**

ROS generation via the NADPH oxidase pathway contributes to invasion of *S. agalactiae* in human endothelial cells accompanied by cytoskeletal reorganisation through the PI3K/Akt pathway, which provides novel evidence for the involvement of oxidative stress in *S. agalactiae* pathogenesis.

*Streptococcus agalactiae* [group B *Streptococcus* (GBS)] is found as a commensal in the gastrointestinal and the genitourinary tracts of up to 30% of healthy adults. Although *S. agalactiae* is a significant cause of neonatal meningitis and septicaemia (Verani et al. 2010), cases of invasive infections have been increasingly reported in elderly and immunocompromised adults, including patients with diabetes mellitus, alcoholism, and cancer (Pimentel et al. 2016).

Pathogenesis of *S. agalactiae* in humans is associated with the bacteria’s ability to invade and pass through anatomic barriers such as vaginal or cervical epithelium. The mechanisms of *S. agalactiae* attachment, invasion, and translocation were investigated in a variety of cellular systems of human origin, including endothelial cells (Buscetta et al. 2016). However, further studies related to the molecular level of pathogen-host cell interactions are required. It is well known that modulation of actin microfilaments is critical for *S. agalactiae* invasion.

Phosphoinositide 3-kinase (PI3K) activation also occurs during the invasive process in host cells by *S. agalactiae* (Burnham et al. 2007). The formation of phosphatidylinositol 3,4,5-triphosphate (PIP_3_) by PI3K leads to Akt phosphorylation and activation on the host-cell membrane. Manipulation of the PI3K/Akt pathway by a pathogen results in coordination of actin rearrangement, which leads to internalisation of the organism. The involvement of PI3K has been observed during the invasion process of *S. agalactiae* and other human pathogens (Burnham et al. 2007, Dowd et al. 2016).

In a previous study, our group showed that the interaction between *S. agalactiae* and human umbilical vein endothelial cells (HUVECs) induced the overexpression of two phosphotyrosine proteins, described as glutathione-S transferase (GST) and annexin V (Santos et al. 2009). Studies have shown that GST protects cells in up to 90% of cases of damage induced by oxidative stress (Awasthi et al. 2017).

Reactive oxygen species (ROS) production by endothelial cells modulates redox-sensitive signalling mechanisms and gene expression that may also induce the activation of the PI3K/Akt pathway (Yang et al. 2017). The major source of ROS in endothelial cells is NADPH oxidase, an O_2_-generating enzyme also expressed in other mammalian cell types (Li and Shah 2003). NADPH oxidase activation (oxidative burst) is essential for immune responses against infection (Ushio-Fukai 2006). A previously published study demonstrated that respiratory oxidative burst was triggered during *S. agalactiae* adherence to macrophages by the activation of NADPH oxidase (Teixeira et al. 2001).

Similarities in the NADPH oxidase complex have been demonstrated between endothelial cells and phagocytes (Li and Shah 2002, Meijles et al. 2014). NADPH oxidase is a multicomponent enzyme that includes two membrane-bound components, gp91*phox* (also known as Nox2) and p22*phox*, and cytosolic subunits, including p40*phox*, p47*phox*, p67*phox*, and the small GTPase Rac1/2 (Abid et al. 2007, Lambeth et al. 2007). Among these subunits, p47*phox* is one of the most important regulators of NADPH oxidase activity. The cytoskeleton of host cells may play a role in the translocation and consequent activation of NADPH oxidase (Touyz et al. 2005, Meijles et al. 2014). However, the molecular mechanisms that promote ROS generation due to *S. agalactiae* infection in human endothelial cells remain unclear. To investigate the influence of oxidative stress on *S. agalactiae*-endothelial cell interactions, we focused on ROS production by activation of the p47*phox* NADPH oxidase subunit and the PI3K/Akt pathway during *S. agalactiae*-endothelial cell interaction.

## MATERIALS AND METHODS

*Bacterial strain origin and culture conditions* - *S. agalactiae* capsular type III [GBS90356 cerebrospinal fluid (CSF) strain] isolated from a 3-day-old male baby with fatal acute meningitis that was partially investigated for virulence properties (Teixeira et al. 2001, Santos et al. 2009, Costa et al. 2011) was used in this study. For experiments described below, the GBS90356 strain was cultured on blood agar base (BAB; Oxoid) plates containing 5% defibrinated sheep’s blood for 24 h at 37ºC and then grown in brain heart infusion broth (BHI; Difco) at 37ºC until reaching an OD_540_ of 0.4 [~10^8^ colony forming units (CFU) per mL] (Santos et al. 2009).

*S. agalactiae-HUVEC interaction assays* - Primary HUVECs were obtained by treating umbilical veins with a 0.1% collagenase IV (Sigma) solution as previously described (Jaffe et al. 1973). All experiments were performed at least three times and each experiment was performed with a pool of HUVECs obtained from different donors. HUVECs were seeded onto 25-cm^2^ bottles coated with porcine skin gelatine (Sigma) and grown in 199 medium (M199 - Sigma) supplemented with antibiotics, 100 U/mL penicillin, 100 μg/mL streptomycin, and 2.5 μg/mL amphotericin-B, 2 mM glutamine, and 20% foetal bovine serum at 37ºC in a humidified 5% CO_2_ atmosphere until they reached confluence. HUVECs grown in M199 without serum (18-24 h) were also used as negative control. Confluent HUVEC monolayers were used during first or second passages only, and subcultures were obtained by treatment with 0.025% trypsin/0.2% EDTA solution prepared in 0.01 M phosphate buffered 0.15 M NaCl at pH 7.2 (PBS), rinsed in serum-depleted culture medium, and used for experiments in 24-well culture plates (Corning, NY, USA) (Santos et al. 2009).

HUVEC monolayers were pre-treated with or without diphenylene iodonium (DPI; 10 µM), an inhibitor of the catalytic subunit of NADPH oxidase or with apocynin (10 µM), a compound that prevents coupling between p47*phox* and the corresponding catalytic subunit, or LY294002, a PI3K inhibitor (5 µM) for 15 min. Then, HUVECs were allowed to interact with *S. agalactiae* (5 × 10^7^ CFU) for various time periods (15, 30, 60, 120, and 180 min) in 5% CO_2_ at 37ºC. Subsequently, infected HUVECs were rinsed three times with PBS and lysed with 0.5 ml of 25 mM Tris, 5 mM EDTA, 150 mM NaCl, and 1% IGEPAL (lysis buffer). The total number of viable bacteria associated (intracellular plus extracellular) to HUVECs was estimated by determining the CFU/mL on blood agar medium. Quantitative determination of intracellular bacteria was performed by a gentamicin-penicillin protection assay, as previously described (Santos et al. 2009). After each incubation period, the infected monolayers were incubated for an additional 2-h period in M199 containing 100 μg/mL gentamicin and 5 μg/mL penicillin G. The number of internalised viable bacteria was assessed as outlined above. Adherence rates were calculated as follows: [CFU of total cell-associated (internalised plus surface adherent) *S. agalactiae* - CFU internalised *S. agalactiae*] (Santos et al. 2009, Costa et al. 2011).

*Measurement of intracellular ROS* - HUVECs were incubated overnight on 96-well black-walled plates at a density of 6 × 10^3^ cells/well, at 37ºC in a humidified atmosphere of 5% CO_2_. The cells were washed three times with PBS and incubated with the probe CM-H2DCFDA [5-(and-6)-chloromethyl-2′,7′-dichlorodihydrofluorescein diacetate] for 30 min. Cells were then washed with PBS and pre-treated with or without either an inhibitor of NADPH oxidase [(DPI; 10 µM) or apocynin (10 µM)], the PI3K inhibitor LY294002 (5 µM), or TNF-α (10 ng/mL; positive control) and incubated in the absence or presence of *S. agalactiae*. Endothelial cells were serum-starved for 18-24 h to exclude serum-dependent effects as a negative control. Fluorescence intensity was assessed throughout the 180-min period using an Envision microplate reader. ROS production was detected through fluorescence emitted from dichlorofluorescein (DCF) oxidation. Fluorescence was monitored at excitation and emission wavelengths of 495 nm and 525 nm, respectively (Ribeiro-Pereira et al. 2014).

*Real time quantitative reverse transcription polymerase chain reaction (Real-time qRT-PCR)* - Total RNA was isolated from samples obtained from HUVEC controls or those infected with *S. agalactiae* by using an RNeasy Mini Kit (Qiagen, Hilden, Germany) according to the manufacturer’s instructions. The isolated mRNA was transcribed into cDNA using SuperScript III First-Strand (Invitrogen, Carlsbad, CA) according to the manufacturer’s protocol. The primers used for p47*phox* were 5′-CCCTGCTGGGCTTTGAGAA-3′ (sense) and 5′-CCGACAGGTCCTGCCATTT-3′ (antisense) (Watanabe et al. 2003). GAPDH was used as the housekeeping gene (endogenous control) for normalisation and was amplified using the following primers: 5′-TGCACCACCAACTGCTTAGC-3′ (sense) and 5′-GCCATGGACTGTGGTCATGAG-3′ (antisense) (Shen et al. 2013). Real Time qRT-PCR was performed using GoTaq qPCR Master Mix reagents (Promega) into a 7500 real-time PCR system thermocycler (Applied Biosystems, Foster City, CA). All real-time PCR reactions were performed in triplicate for each independent experiment. Data were normalised to GAPDH expression and relative expression was calculated by the 2-ΔΔCT comparative method (Bülbül et al. 2014).

*Immunoblot analysis* - HUVEC monolayers were infected with *S. agalactiae* for different time periods as described above. Following infection, the plates were chilled, and all subsequent steps were carried out at 4ºC as previously described (Costa et al. 2016). The HUVECs were then rinsed with PBS containing 0.4 mM Na_3_VO_4_ and 1 mMNaF per mL. Next, the infected cells were scraped from the plate, resuspended in 1.5 mL of the same buffer solution, collected by centrifugation for 1 min at 12,000 ´ *g*, and lysed for 30 min in 100 µL of 50 mM Tris-HCL (pH 7.6) containing 0.4 mM Na_3_VO_4_, 1 mM NaF, 1% Triton X-100, 100 µM of phenylethylsulfonyl fluoride, 40 µM of leupeptin, and 2 mM EDTA. The proteins were then quantified, and 30 µg of protein from each extract was subjected to electrophoresis in 12% polyacrylamide separating gel (SDS-PAGE). Proteins were transferred to nitrocellulose membranes (BioRad), which were blocked and then incubated with polyclonal anti-human antibodies: phospho-p47*phox*, β-actin, total Akt, and phospho-Akt. The membranes were incubated with the appropriate peroxidase-conjugated secondary antibody and immunoreactivity was detected using an ECL Plus detection kit (Amersham Biosciences, Buckinghamshire, UK). Autoradiographs were quantified by scanning densitometry, and the resulting absorbance curves were integrated using the Scion Image Master. Densitometric analyses were performed on gels with different exposure times, and the ones giving linear absorbance curves were used for semiquantitative assessment (Santos et al. 2013).

*Immunofluorescence* - HUVECs were cultured on glass coverslips then pre-treated or not with DPI (10 µM), apocynin (10 µM), or LY294002 (5 µM) for 15 min and then allowed to interact with fluorescein-isothiocyanate (FITC)-labelled *S. agalactiae* for 60 min at 37ºC. The cells were washed with PBS, fixed in 3.7% formaldehyde for 10 min at 25º and washed again 3 times with PBS-BSA (10 min each). Cells were permeabilised with 0.1% Triton X-100 in PBS for 6 min, washed, and stained with 0.1 μg/mL fluorescein isothiocyanate-phalloidin, 0.5 μg/mL 4′-6-diamidino-2-phenylindole (DAPI) or anti-phosphorylated *p47phox* Alexa 546 conjugated for 30 min. Cells were visualised in an Olympus Microscope Model IX71 TH4-100 (Santos et al. 2009).

*Statistical analysis* - The values of different treatments were compared using Student’s *t*-test and ANOVA, followed by Bonferroni’s *t*-test for unpaired values. All statistical analyses were performed at the p < 0.05 level of significance.

## RESULTS

**Fig. 1:**
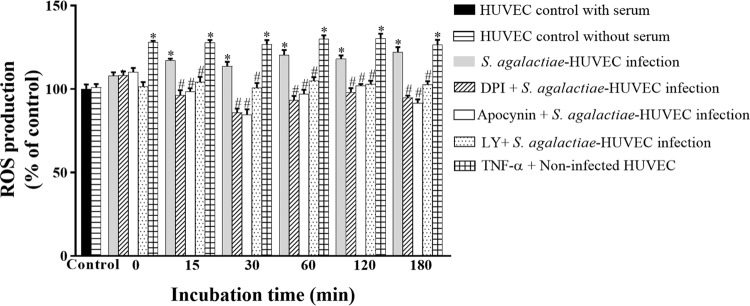
: reactive oxygen species (ROS) generation by NADPH oxidase activity during infection of human primary endothelial cells (HUVECs) by *Streptococcus agalactiae*. Experiments were performed at different periods of incubation using the GBS90356 type III strain of *S. agalactiae* and HUVECs in the presence or absence of NADPH inhibitors (10 μΜ DPI, 10 μΜ apocynin) or a PI3K inhibitor (5 μΜ LY294002). Non-infected HUVEC monolayers with and without serum were used as negative controls. Non-infected HUVEC monolayers with serum and 10 ng/mL TNF-α were used as positive controls. Data are expressed as mean ± SD of three independent experiments; *p ≤ 0.05 vs non-infected HUVECs; #p ≤ 0.05 vs *S. agalactiae*-HUVEC infection.

*ROS production during S. agalactiae-HUVEC interaction* - Levels of ROS generation were evaluated during *S. agalactiae-*HUVEC interaction using inhibitors of NADPH oxidase (DPI and apocynin) and PI3K (LY294002). Non-infected HUVEC monolayers treated with TNF-α were used as positive controls. Increased ROS production was observed in HUVECs after 15 min of infection (Fig. 1; p < 0.02). Moreover, pre-treatment of HUVEC with all inhibitors decreased ROS production during all time periods (p < 0.03). HUVECs grown in serum starvation conditions for 18-24 h to exclude serum-dependent effects were used as a negative control.

**Fig. 2:**
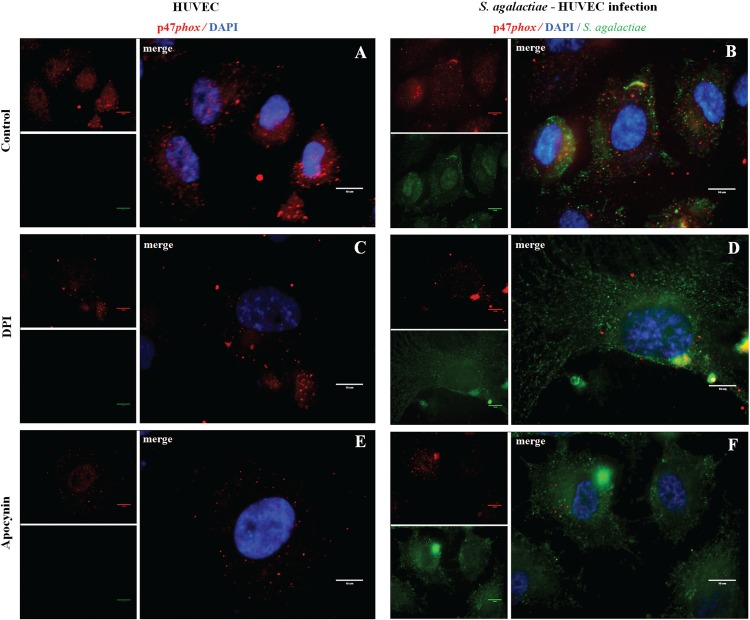
: immunofluorescence assays of NADPH oxidase p47*phox* subunit activity during infection of human primary endothelial cells (HUVECs) by *Streptococcus agalactiae*. (A, C, E) Uninfected HUVECs and (B, D, F) HUVECs infected by *S. agalactiae* in the presence or absence of NADPH inhibitors (10 μΜ DPI, 10 μΜ apocynin). Anti-p47*phox* was labelled with Alexa 546 (red), *S. agalactiae* with fluorescein-isothiocyanate (FITC) (green), and the nuclei were stained with DAPI (blue). Scale bars, 10 μM.

*Activation of p47phox NADPH oxidase subunit during S. agalactiae-HUVEC interaction* - Results of immunofluorescence assays of p47*phox* activity during ROS generation by *S. agalactiae* infection of HUVEC were demonstrated in Fig. 2A-F. The NADPH oxidase p47*phox* subunit was mostly observed near the nucleus of uninfected host cells (Fig. 2A). HUVECs infected with *S. agalactiae* showed changes in subcellular localisation of the p47*phox* subunit, which was previously shown to be central for even distribution in the cell, reflecting activation of p47*phox* in HUVECs (Fig. 2B). Additional assays are required to elucidate mechanisms involved in p47*phox* activation by *S. agalactiae*. DPI and apocynin inhibition assays (Fig. 2C-F) were performed at 60 min post-infection when the highest adherence level to HUVECs by *S. agalactiae* was observed. Treatment with both inhibitors resulted in higher adherence levels of *S. agalactiae* 60 min post-infection of in HUVECs (Fig. 2D, F).

**Fig. 3:**
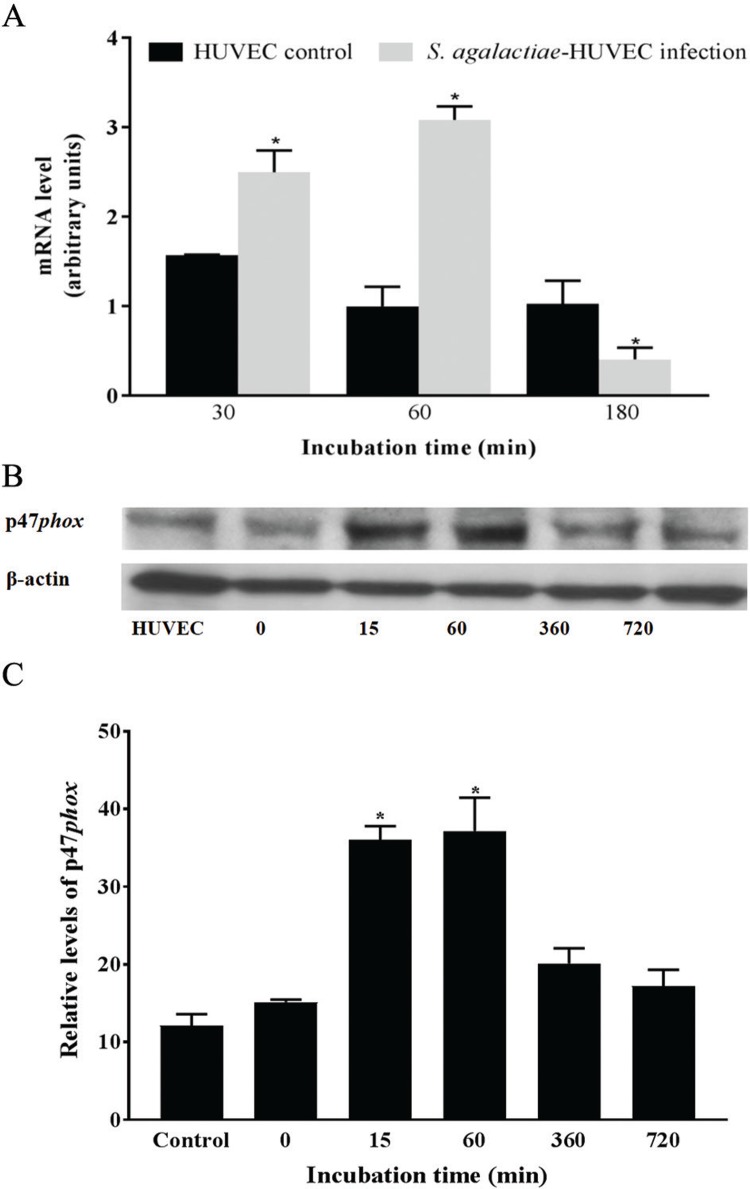
: expression of NADPH oxidase p47*phox* subunit during infection of HUVECs with *Streptococcus agalactiae*. Data were analysed at different periods of time by (A) real time quantitative reverse transcription polymerase chain reaction (real-time qRT-PCR), (B) immunoblotting, and (C) densitometry assays. Actin was used as a control for protein loading. Data are expressed as mean ± SD of three experiments; *p < 0.05.

*NADPH oxidase p47phox expression during S. agalactiae-HUVEC* - Data displayed in Fig. 3A show an increase of p47*phox* mRNA expression at 30 min and 60 min post-infection, as measured by Real Time qRT-PCR (p < 0.03). Immunoblotting assay results confirmed higher p47*phox* activities at upon 15 min and 60 min incubation of *S. agalactiae*-HUVEC (p < 0.04) (Fig. 3B, C).

**Fig. 4:**
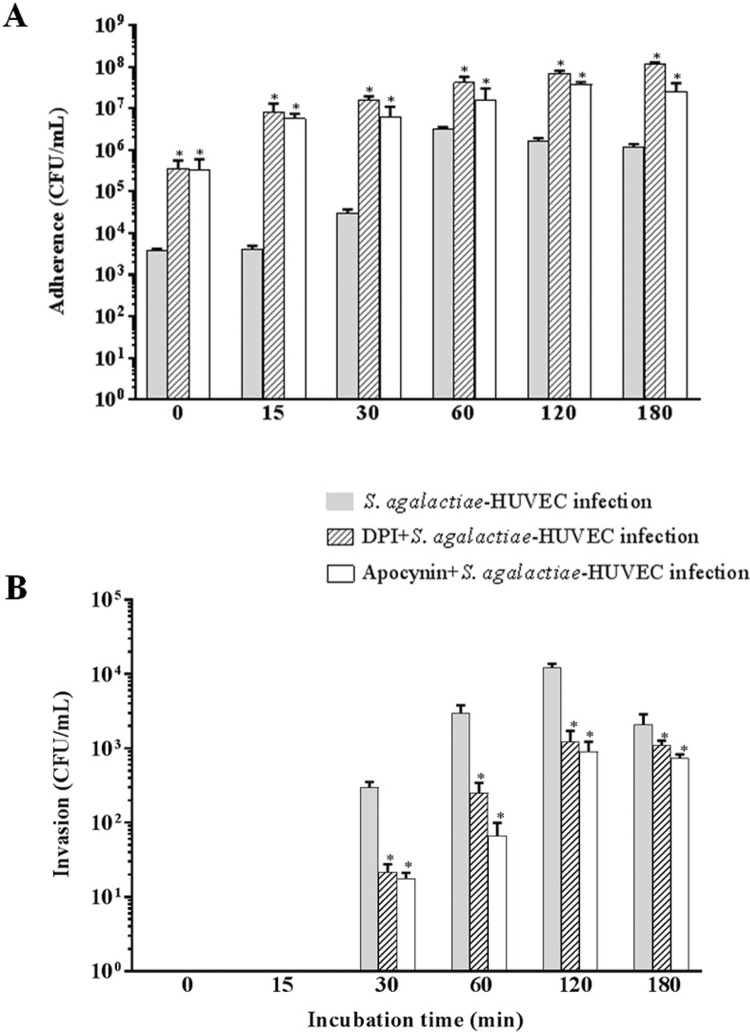
: influence of NADPH oxidase *p47phox* subunit on *Streptococcus agalactiae*-human primary endothelial cells (HUVECs) interaction. (A) Adherence to and (B) intracellular viability of *S. agalactiae* in HUVECs pre-treated with DPI (10 µM) or apocynin (10 µM) to inhibit p47*phox*. Data are expressed as mean ± SD of three experiments; *p < 0.05.

*Effect of DPI and Apocynin on activation of the p47phox NADPH oxidase subunit during S. agalactiae-HUVEC interaction* - The influence of the NADPH oxidase p47*phox* subunit on *S. agalactiae* adherence to and intracellular viability of HUVEC using inhibitors of p47*phox* (DPI or apocynin) are displayed in Fig. 4. A time-dependent increase in adherence to HUVECs was observed upon treatment with both inhibitors. A higher number of adherent bacteria was observed in HUVECs pre-treated with DPI or apocynin, compared to that of untreated HUVECs (p < 0.03) (Fig. 4A). A significant decrease in the number of internalised bacteria occurred in HUVECs treated with DPI and apocynin after the first 30 min of infection compared to the number in untreated HUVECs (Fig. 4B).

**Fig. 5:**
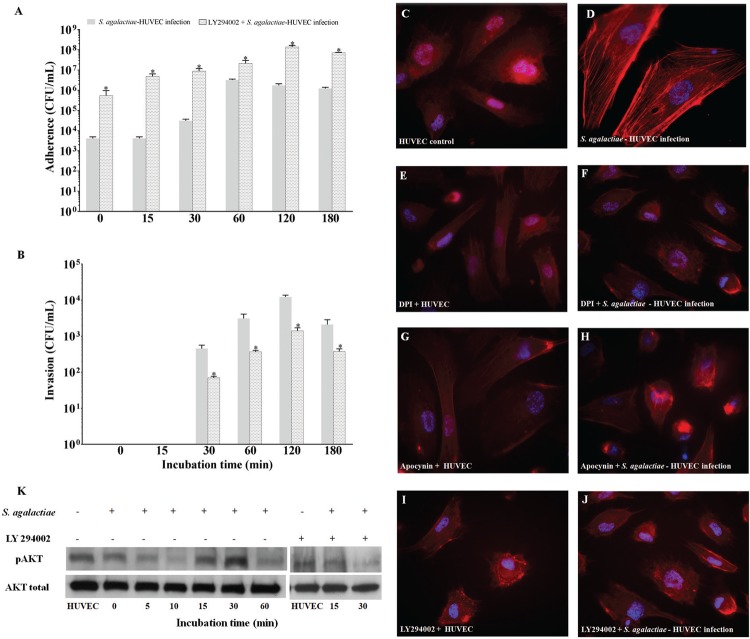
: influence of the PI3K/Akt pathway on the *Streptococcus agalactiae*-human primary endothelial cells (HUVECs) interaction. (A) Adherence to and (B) invasion of *S. agalactiae* in HUVECs pre-treated with LY294002, an inhibitor of PI3K. Micrographic images of immunofluorescence staining indicated: (C) non-infected HUVEC monolayers as a negative control; (D) generation of actin stress fibres in HUVECs infected with *S. agalactiae*; (E, G, I) HUVECs pre-treated with DPI (10 µM), apocynin (10 µM), and LY294002 (5 µM), respectively; (F, H, J) HUVECs pre-treated with inhibitors (DPI, apocynin and LY294002, respectively) and infected with *S. agalactiae*. (K) Immunoblotting assay of phosphorylated Akt in infected HUVECs pre-treated with LY294002. Data are expressed as mean ± SD of three experiments. *p < 0.05.

*Involvement of the PI3K/Akt pathway during S. agalactiae-HUVEC interaction* - Data shown in Fig. 5A-B demonstrate the effect of the PI3K/Akt pathway on the interaction between *S. agalactiae* and HUVECs pre-treated with the PI3K inhibitor, LY294002. A time-dependent increase in bacterial adherence was observed when HUVECs were treated with LY294002, similarly to results achieved with p47*phox* (DPI and apocynin) inhibitors. The highest adherence level was observed at 120 min post-infection (p < 0.02) (Fig. 5A). Conversely, bacterial internalisation was detected at lower levels from 30 min to 180 min post-infection (p < 0.01) (Fig. 5B).

Immunofluorescence staining assays illustrated the involvement of PI3K/Akt pathway activation in the actin stress fibre assembly of endothelial cells upon by *S. agalactiae* infection (Fig. 5C-D). Similar to non-infected HUVECs, pre-treatment with NADPH oxidase (DPI and apocynin) or PI3K (LY294002) inhibitors in the presence (Fig. 5F, H, J) or absence of *S. agalactiae* infection (Fig. 5E, G, I) did not induce actin stress fibre arrangement. As shown in Fig. 5K, immunoblotting results revealed higher levels of phosphorylated Akt expression at 15 min and 30 min post-infection of HUVECs by *S. agalactiae*. Inhibition assays with LY294002 (15 min and 30 min post-infection) confirmed the involvement of the PI3K/AKT pathway during *S. agalactiae* internalisation in endothelial cells. Results were confirmed by densitometry analysis (data not shown).

## DISCUSSION

ROS have been classically regarded as host defence molecules related to destroying exogenous human pathogens. Also, it has long been recognised that increased ROS levels modify cell signalling of host proteins, leading to pathological processes such as inflammation and bacterial infections. Nevertheless, accumulated evidence indicates that ROS are second messengers and cell signalling modifiers (Zhang et al. 2016).

Only a few studies concerning NADPH oxidase activation during *Streptococcus* spp*.* infection were found in the available literature. For *Streptococcus pyogenes*, the production of O_2_^-^ was found as the result of NADPH oxidase induction during bacterial infection in human primary keratinocytes (Regnier et al. 2016). The effect of NADPH oxidase inhibitor (DPI) treatment on intracellular survival of *S. pyogenes* during the early stage of phagocytosis by HL-60 cells was also verified (Nordenfelt et al. 2009). Moreover, the ability of *S. agalactiae* to trigger oxidative burst in murine macrophages by NADPH oxidase activation was also demonstrated (Teixeira et al. 2001). In addition, the role of p47*phox* in functionally active NADPH oxidase activity was evidenced by using p47*phox* ^*-/-*^ mice assays (Li and Shah 2002, Touyz et al. 2005). Similar effects were demonstrated for endothelial cells: the major source of ROS was also described as NADPH oxidase, whose activation and regulation were controlled by the phosphorylation of its cytosolic component p47*phox* (Schuett et al. 2017). However, physiological substrates, regulation, and control of signalling networks, especially of endothelial cells, need further investigation.

Presently, the involvement of p47*phox* subunit in *S. agalactiae*-HUVEC interaction was demonstrated by Real Time qRT-PCR, western blotting, and immunofluorescence assays. ROS production by activation of the p47*phox* NADPH oxidase subunit during *S. agalactiae*-HUVEC interaction was demonstrated. Thus, our results agree with those of Zhang et al. (2016) showing that DPI and apocynin reduce p47*phox* translocation and expression, thereby suppressing ROS production in peripheral blood mononuclear cells from premature infants. Similarly, *Streptococcus pneumoniae* induces a higher oxidative burst in neutrophils by triggering neutrophil NADPH oxidase to produce more reactive oxygen intermediates and by activating p47*phox* by *S. pneumoniae* in the plasma membrane fraction of neutrophils (Barbuti et al. 2010).

It is well-known that *S. agalactiae* needs cytoskeleton reorganisation to invade host cells (Boone et al. 2012). Interestingly, previous studies have also reported the involvement of ROS in cytoskeletal reorganisation accompanying O_2_^-^ formation acting as a scaffold for the p47*phox* subunit (Touyz et al. 2005). A close relationship between actin polymerisation and ROS generation has also been observed after wounding endothelial cell monolayers (Li and Shah 2002).

The PI3K/Akt signalling pathway was found to be associated with cytoskeletal regulation, vesicle trafficking, and the balance between cellular survival and regulated cell death (Burnham et al. 2007). PI3K activation has been specifically implicated in modification and manipulation of the actin cytoskeleton, essentially in *S. agalactiae* invasion of host cells (Burnham et al. 2007). The involvement of the PI3K/Akt pathway during the *S. agalactiae*-HUVEC interaction was verified in the present study. Burnham et al. (2007) showed an inhibition of *S. agalactiae* invasion in HeLa cells treated with the LY294002 inhibitor of PI3K. Similar to previous results of these authors, current data concerning the inhibition of ROS production showed a decrease in invasion and an increase in the adhesion levels of *S. agalactiae* on HUVEC induced by LY294002. Data indicated that *S. agalactiae*-HUVEC interaction was partially due to alterations in the organisation of microfilaments of the host cells through the PI3K/Akt pathway. As soon as *S. agalactiae* began to establish physical contact with the host cell surface, the formation of stress fibres was observed in HUVECs. Additional results demonstrated loss of stress fibres due to inhibition of PI3K, reinforcing the participation of the PI3K/Akt pathway in human endothelial cells.

The ability of Akt to regulate multiple activities of host cells makes it an essential target for invading bacterial pathogens, including *S. agalactiae*. A previous study in HeLa cells indicated that Akt was required for efficient *S. agalactiae* invasion, since Akt was rapidly phosphorylated in response to *S. agalactiae* (Burnham et al. 2007). In our work using HUVECs, a significant increase in Akt phosphorylation occurred at 15-30 min post-infection of *S. agalactiae*, suggesting the involvement of PI3K/Akt in this process.

In conclusion, ROS generation via the NADPH oxidase pathway in human endothelial cells accompanied by cytoskeletal reorganisation through the PI3K/Akt pathway occurred during invasion of the *S. agalactiae* GBS90356 strain isolated from a fatal case of meningitis. These results may contribute to the understanding of virulence mechanisms involved in the translocation of *S. agalactiae* across the blood-brain barrier.
